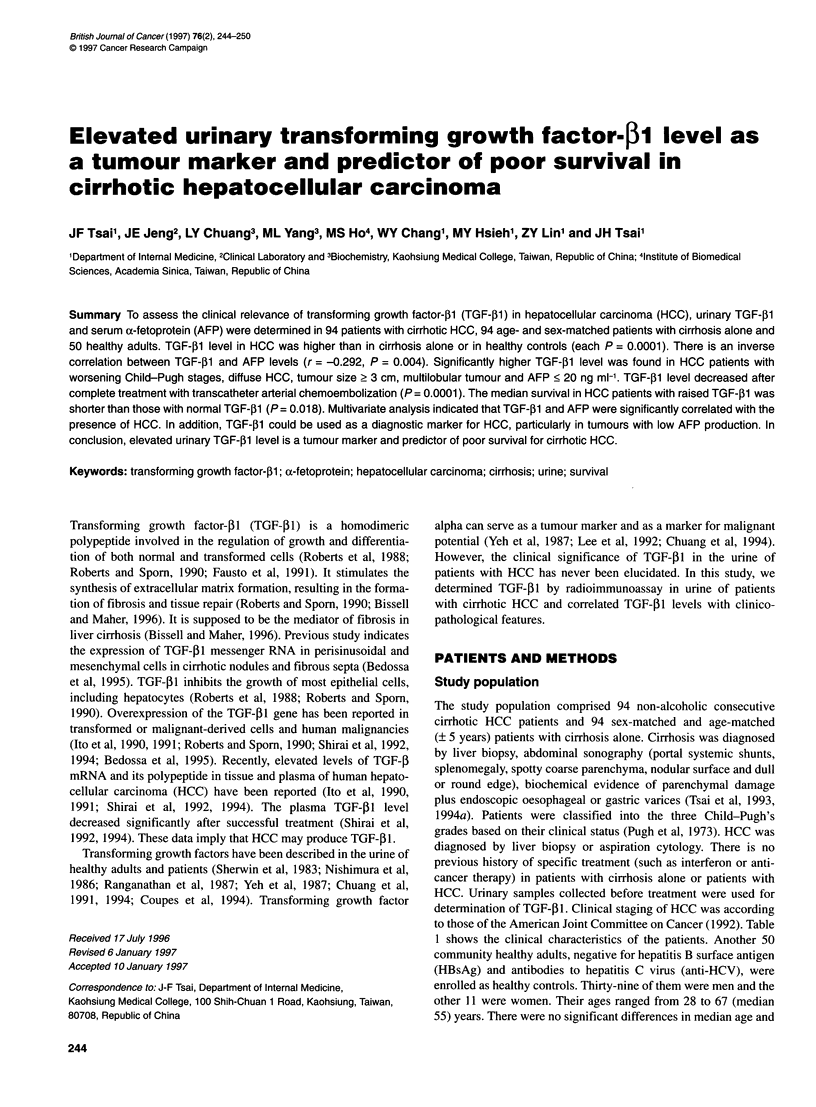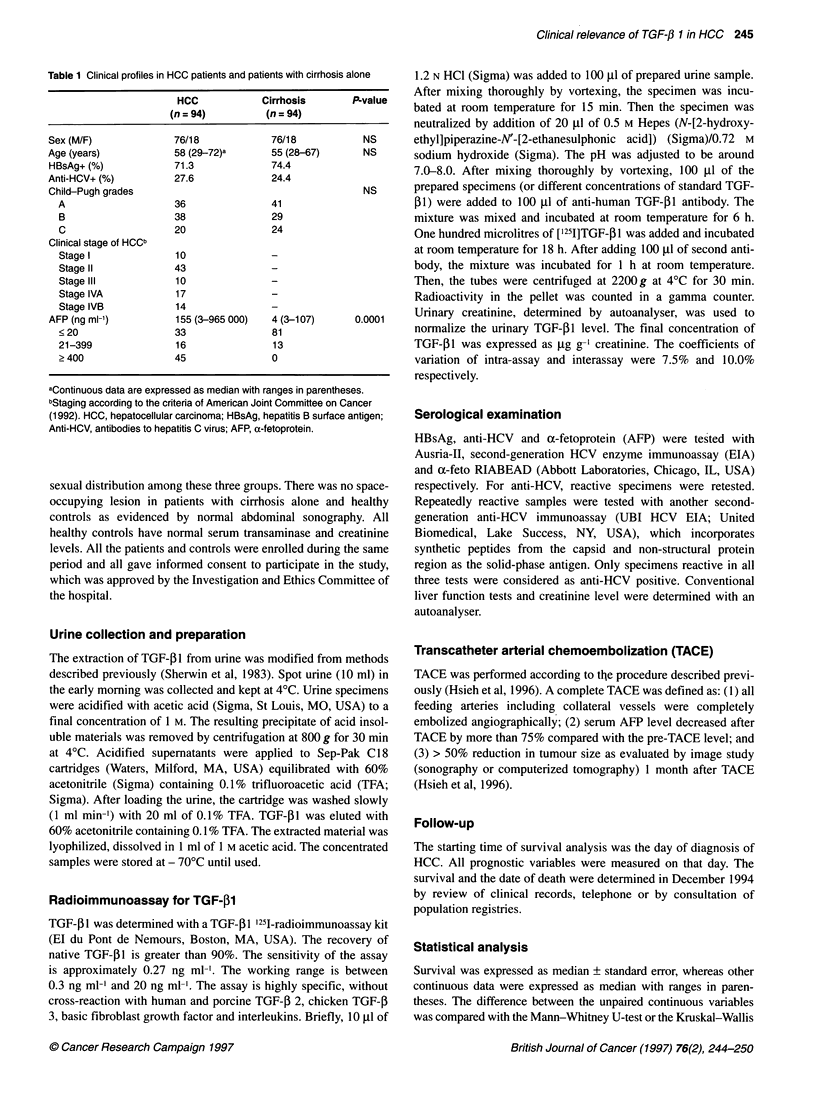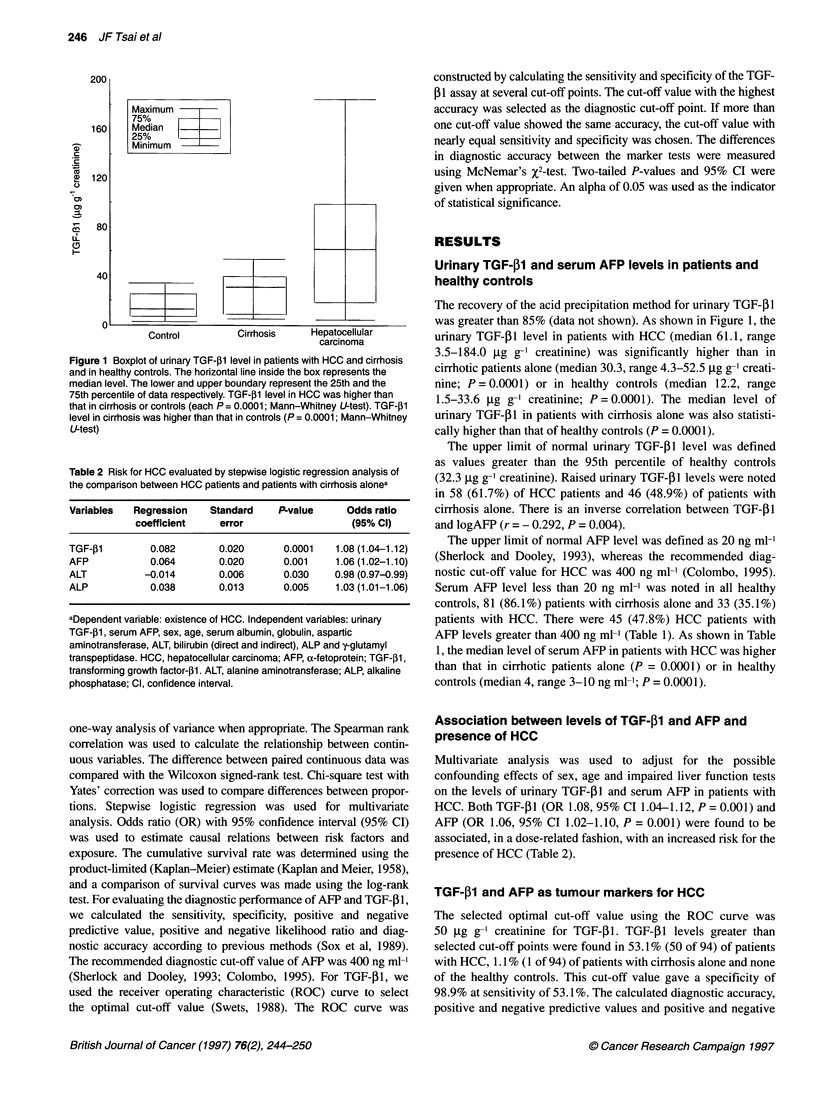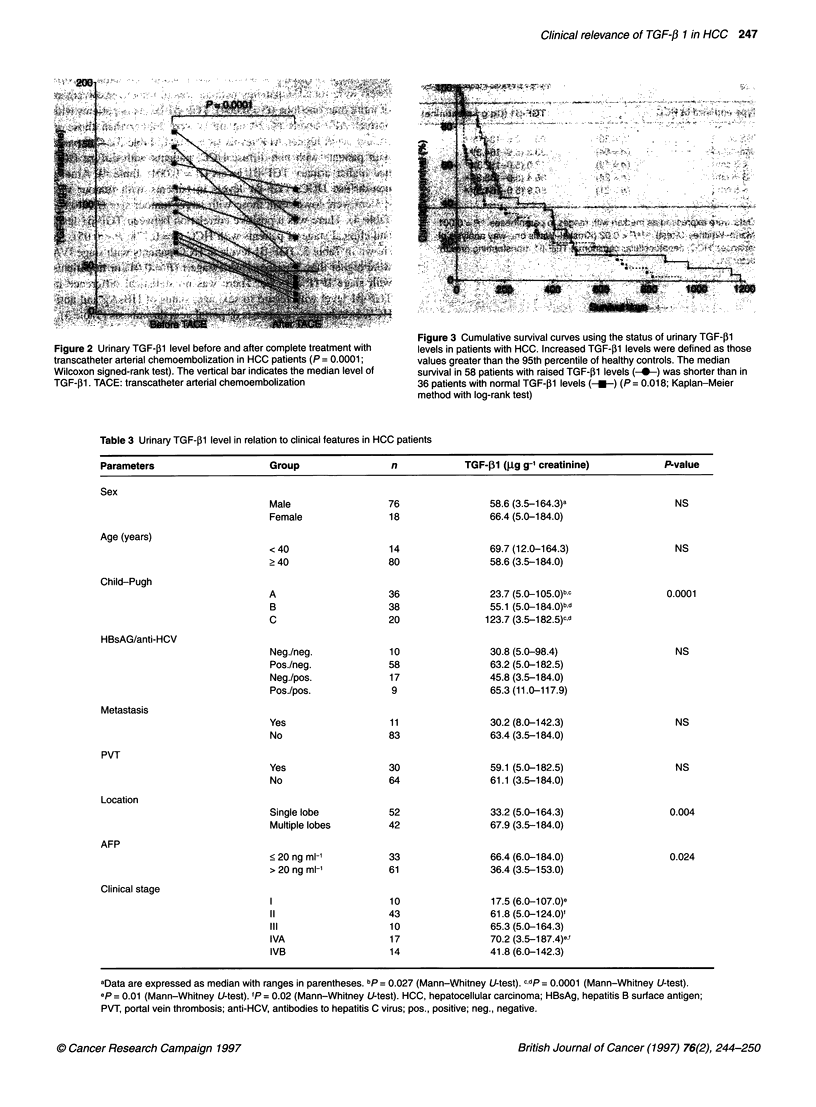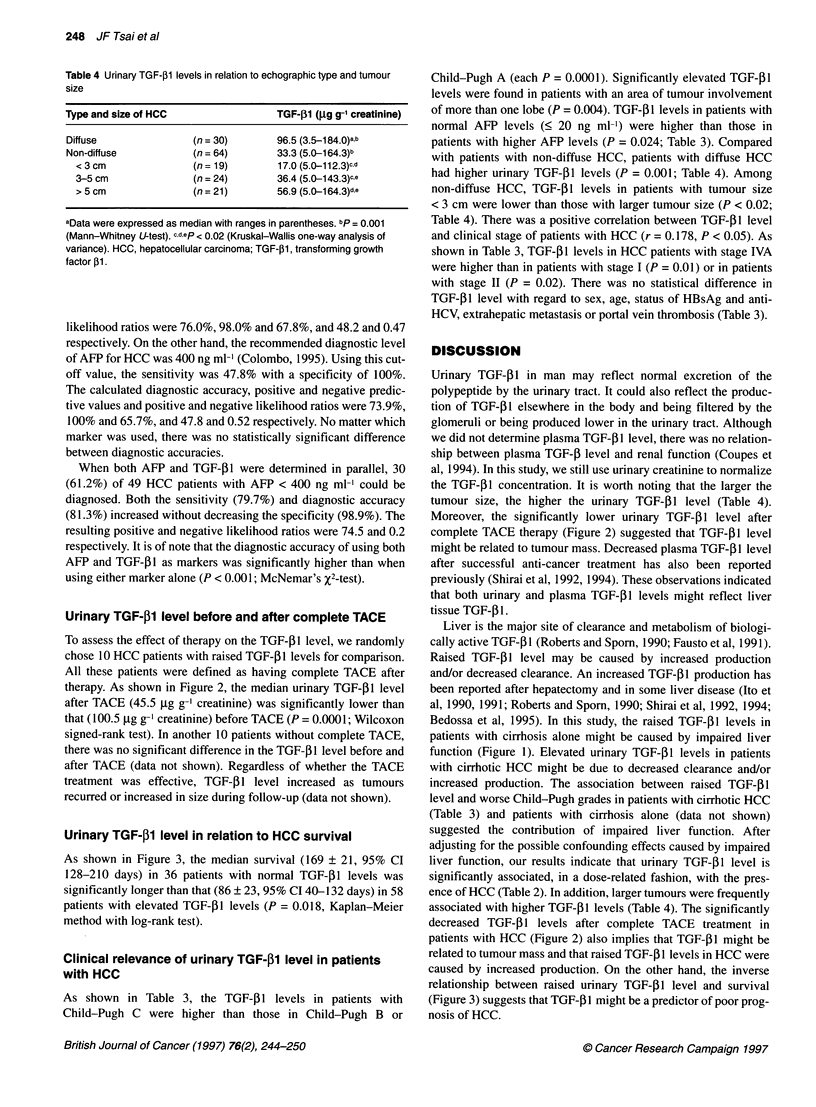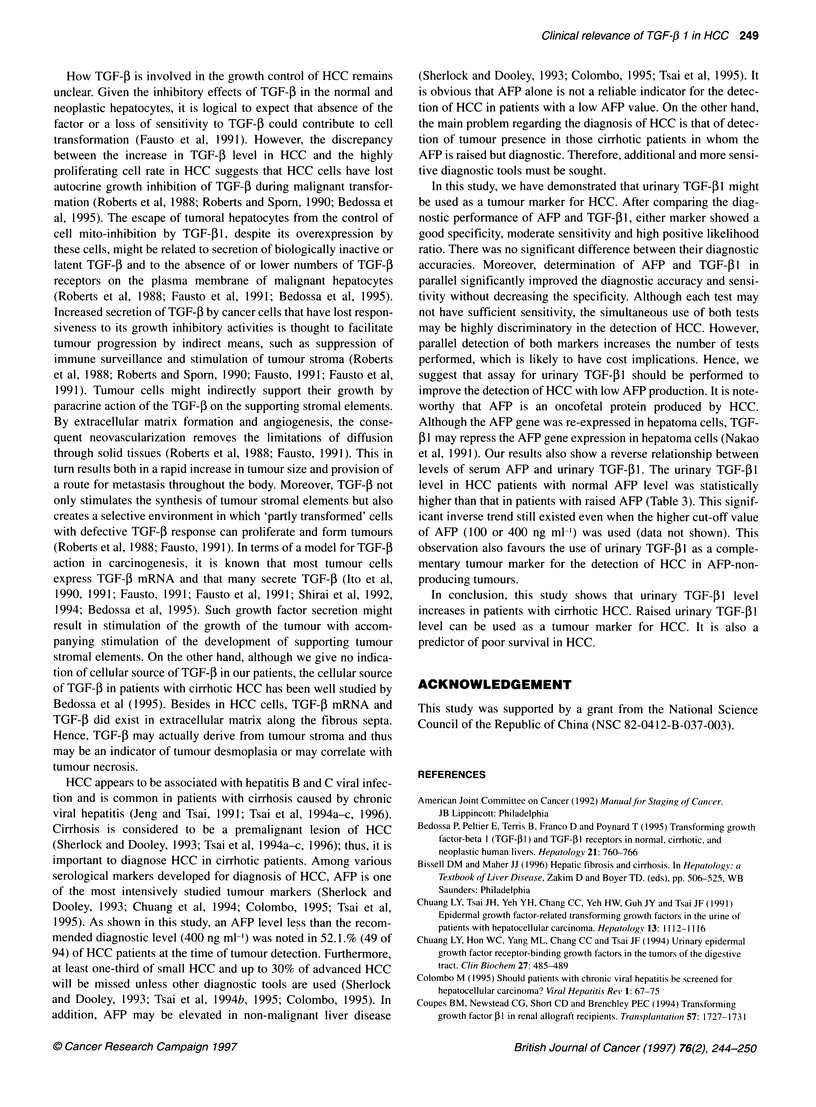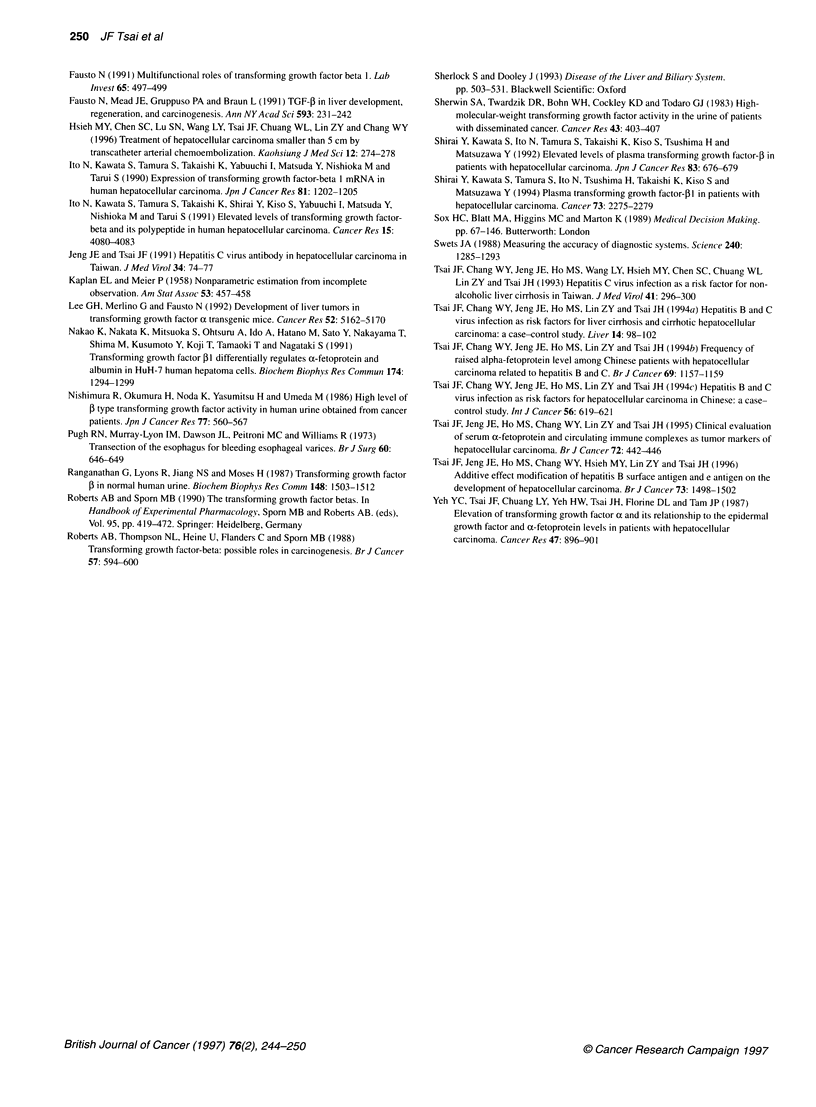# Elevated urinary transforming growth factor-beta1 level as a tumour marker and predictor of poor survival in cirrhotic hepatocellular carcinoma.

**DOI:** 10.1038/bjc.1997.369

**Published:** 1997

**Authors:** J. F. Tsai, J. E. Jeng, L. Y. Chuang, M. L. Yang, M. S. Ho, W. Y. Chang, M. Y. Hsieh, Z. Y. Lin, J. H. Tsai

**Affiliations:** Department of Internal Medicine, Kaohsiung Medical College, Taiwan, Republic of China.

## Abstract

To assess the clinical relevance of transforming growth factor-beta1 (TGF-beta1) in hepatocellular carcinoma (HCC), urinary TGF-beta1 and serum alpha-fetoprotein (AFP) were determined in 94 patients with cirrhotic HCC, 94 age- and sex-matched patients with cirrhosis alone and 50 healthy adults. TGF-beta1 level in HCC was higher than in cirrhosis alone or in healthy controls (each P = 0.0001). There is an inverse correlation between TGF-beta1 and AFP levels (r = -0.292, P = 0.004). Significantly higher TGF-beta1 level was found in HCC patients with worsening Child-Pugh stages, diffuse HCC, tumour size > 3 cm, multilobular tumour and AFP < or = 20 ng ml(-1). TGF-beta1 level decreased after complete treatment with transcatheter arterial chemoembolization (P = 0.0001). The median survival in HCC patients with raised TGF-beta1 was shorter than those with normal TGF-beta1 (P = 0.018). Multivariate analysis indicated that TGF-beta1 and AFP were significantly correlated with the presence of HCC. In addition, TGF-beta1 could be used as a diagnostic marker for HCC, particularly in tumours with low AFP production. In conclusion, elevated urinary TGF-beta1 level is a tumour marker and predictor of poor survival for cirrhotic HCC.


British Journal of Cancer (1997) 76(2), 244–250
© 1997 Cancer Research Campaign

### Elevated urinary transforming growth factor-β1 level as a tumour marker and predictor of poor survival in cirrhotic hepatocellular carcinoma

**JF Tsai[1], JE Jeng[2], LY Chuang[3], ML Yang[3], MS Ho[4], WY Chang[1], MY Hsieh[1], ZY Lin[1] and JH Tsai[1]**

[1]Department of Internal Medicine, [2]Clinical Laboratory and [3]Biochemistry, Kaohsiung Medical College, Taiwan, Republic of China; [4]Institute of Biomedical Sciences, Academia Sinica, Taiwan, Republic of China

**Summary** To assess the clinical relevance of transforming growth factor-β1 (TGF-β1) in hepatocellular carcinoma (HCC), urinary TGF-β1 and serum α-fetoprotein (AFP) were determined in 94 patients with cirrhotic HCC, 94 age- and sex-matched patients with cirrhosis alone and 50 healthy adults. TGF-β1 level in HCC was higher than in cirrhosis alone or in healthy controls (each $P = 0.0001$). There is an inverse correlation between TGF-β1 and AFP levels ($r = -0.292$, $P = 0.004$). Significantly higher TGF-β1 level was found in HCC patients with worsening Child–Pugh stages, diffuse HCC, tumour size ≥ 3 cm, multilobular tumour and AFP ≤ 20 ng $ml^{-1}$. TGF-β1 level decreased after complete treatment with transcatheter arterial chemoembolization ($P = 0.0001$). The median survival in HCC patients with raised TGF-β1 was shorter than those with normal TGF-β1 ($P = 0.018$). Multivariate analysis indicated that TGF-β1 and AFP were significantly correlated with the presence of HCC. In addition, TGF-β1 could be used as a diagnostic marker for HCC, particularly in tumours with low AFP production. In conclusion, elevated urinary TGF-β1 level is a tumour marker and predictor of poor survival for cirrhotic HCC.

**Keywords:** transforming growth factor-β1; α-fetoprotein; hepatocellular carcinoma; cirrhosis; urine; survival

Transforming growth factor-β1 (TGF-β1) is a homodimeric polypeptide involved in the regulation of growth and differentiation of both normal and transformed cells (Roberts et al, 1988; Roberts and Sporn, 1990; Fausto et al, 1991). It stimulates the synthesis of extracellular matrix formation, resulting in the formation of fibrosis and tissue repair (Roberts and Sporn, 1990; Bissell and Maher, 1996). It is supposed to be the mediator of fibrosis in liver cirrhosis (Bissell and Maher, 1996). Previous study indicates the expression of TGF-β1 messenger RNA in perisinusoidal and mesenchymal cells in cirrhotic nodules and fibrous septa (Bedossa et al, 1995). TGF-β1 inhibits the growth of most epithelial cells, including hepatocytes (Roberts et al, 1988; Roberts and Sporn, 1990). Overexpression of the TGF-β1 gene has been reported in transformed or malignant-derived cells and human malignancies (Ito et al, 1990, 1991; Roberts and Sporn, 1990; Shirai et al, 1992, 1994; Bedossa et al, 1995). Recently, elevated levels of TGF-β mRNA and its polypeptide in tissue and plasma of human hepatocellular carcinoma (HCC) have been reported (Ito et al, 1990, 1991; Shirai et al, 1992, 1994). The plasma TGF-β1 level decreased significantly after successful treatment (Shirai et al, 1992, 1994). These data imply that HCC may produce TGF-β1.

Transforming growth factors have been described in the urine of healthy adults and patients (Sherwin et al, 1983; Nishimura et al, 1986; Ranganathan et al, 1987; Yeh et al, 1987; Chuang et al, 1991, 1994; Coupes et al, 1994). Transforming growth factor alpha can serve as a tumour marker and as a marker for malignant potential (Yeh et al, 1987; Lee et al, 1992; Chuang et al, 1994). However, the clinical significance of TGF-β1 in the urine of patients with HCC has never been elucidated. In this study, we determined TGF-β1 by radioimmunoassay in urine of patients with cirrhotic HCC and correlated TGF-β1 levels with clinicopathological features.

*Received 17 July 1996*
*Revised 6 January 1997*
*Accepted 10 January 1997*

*Correspondence to:* J-F Tsai, Department of Internal Medicine, Kaohsiung Medical College, 100 Shih-Chuan 1 Road, Kaohsiung, Taiwan, 80708, Republic of China

### PATIENTS AND METHODS

### Study population

The study population comprised 94 non-alcoholic consecutive cirrhotic HCC patients and 94 sex-matched and age-matched (± 5 years) patients with cirrhosis alone. Cirrhosis was diagnosed by liver biopsy, abdominal sonography (portal systemic shunts, splenomegaly, spotty coarse parenchyma, nodular surface and dull or round edge), biochemical evidence of parenchymal damage plus endoscopic oesophageal or gastric varices (Tsai et al, 1993, 1994*a*). Patients were classified into the three Child–Pugh's grades based on their clinical status (Pugh et al, 1973). HCC was diagnosed by liver biopsy or aspiration cytology. There is no previous history of specific treatment (such as interferon or anticancer therapy) in patients with cirrhosis alone or patients with HCC. Urinary samples collected before treatment were used for determination of TGF-β1. Clinical staging of HCC was according to those of the American Joint Committee on Cancer (1992). Table 1 shows the clinical characteristics of the patients. Another 50 community healthy adults, negative for hepatitis B surface antigen (HBsAg) and antibodies to hepatitis C virus (anti-HCV), were enrolled as healthy controls. Thirty-nine of them were men and the other 11 were women. Their ages ranged from 28 to 67 (median 55) years. There were no significant differences in median age and

**Table 1** Clinical profiles in HCC patients and patients with cirrhosis alone

| | HCC ($n$ = 94) | Cirrhosis ($n$ = 94) | $P$-value |
|---|---|---|---|
| Sex (M/F) | 76/18 | 76/18 | NS |
| Age (years) | 58 (29–72)[a] | 55 (28–67) | NS |
| HBsAg+ (%) | 71.3 | 74.4 | |
| Anti-HCV+ (%) | 27.6 | 24.4 | |
| Child–Pugh grades | | | NS |
| A | 36 | 41 | |
| B | 38 | 29 | |
| C | 20 | 24 | |
| Clinical stage of HCC[b] | | | |
| Stage I | 10 | – | |
| Stage II | 43 | – | |
| Stage III | 10 | – | |
| Stage IVA | 17 | – | |
| Stage IVB | 14 | – | |
| AFP (ng ml$^{-1}$) | 155 (3–965 000) | 4 (3–107) | 0.0001 |
| ≤ 20 | 33 | 81 | |
| 21–399 | 16 | 13 | |
| ≥ 400 | 45 | 0 | |

[a]Continuous data are expressed as median with ranges in parentheses. [b]Staging according to the criteria of American Joint Committee on Cancer (1992). HCC, hepatocellular carcinoma; HBsAg, hepatitis B surface antigen; Anti-HCV, antibodies to hepatitis C virus; AFP, α-fetoprotein.

sexual distribution among these three groups. There was no space-occupying lesion in patients with cirrhosis alone and healthy controls as evidenced by normal abdominal sonography. All healthy controls have normal serum transaminase and creatinine levels. All the patients and controls were enrolled during the same period and all gave informed consent to participate in the study, which was approved by the Investigation and Ethics Committee of the hospital.

### Urine collection and preparation

The extraction of TGF-β1 from urine was modified from methods described previously (Sherwin et al, 1983). Spot urine (10 ml) in the early morning was collected and kept at 4°C. Urine specimens were acidified with acetic acid (Sigma, St Louis, MO, USA) to a final concentration of 1 M. The resulting precipitate of acid insoluble materials was removed by centrifugation at 800 $g$ for 30 min at 4°C. Acidified supernatants were applied to Sep-Pak C18 cartridges (Waters, Milford, MA, USA) equilibrated with 60% acetonitrile (Sigma) containing 0.1% trifluoroacetic acid (TFA; Sigma). After loading the urine, the cartridge was washed slowly (1 ml min$^{-1}$) with 20 ml of 0.1% TFA. TGF-β1 was eluted with 60% acetonitrile containing 0.1% TFA. The extracted material was lyophilized, dissolved in 1 ml of 1 M acetic acid. The concentrated samples were stored at – 70°C until used.

### Radioimmunoassay for TGF-β1

TGF-β1 was determined with a TGF-β1 $^{125}$I-radioimmunoassay kit (EI du Pont de Nemours, Boston, MA, USA). The recovery of native TGF-β1 is greater than 90%. The sensitivity of the assay is approximately 0.27 ng ml$^{-1}$. The working range is between 0.3 ng ml$^{-1}$ and 20 ng ml$^{-1}$. The assay is highly specific, without cross-reaction with human and porcine TGF-β 2, chicken TGF-β 3, basic fibroblast growth factor and interleukins. Briefly, 10 μl of 1.2 N HCl (Sigma) was added to 100 μl of prepared urine sample. After mixing thoroughly by vortexing, the specimen was incubated at room temperature for 15 min. Then the specimen was neutralized by addition of 20 μl of 0.5 M Hepes ($N$-[2-hydroxyethyl]piperazine-$N'$-[2-ethanesulphonic acid]) (Sigma)/0.72 M sodium hydroxide (Sigma). The pH was adjusted to be around 7.0–8.0. After mixing thoroughly by vortexing, 100 μl of the prepared specimens (or different concentrations of standard TGF-β1) were added to 100 μl of anti-human TGF-β1 antibody. The mixture was mixed and incubated at room temperature for 6 h. One hundred microlitres of [$^{125}$I]TGF-β1 was added and incubated at room temperature for 18 h. After adding 100 μl of second antibody, the mixture was incubated for 1 h at room temperature. Then, the tubes were centrifuged at 2200 $g$ at 4°C for 30 min. Radioactivity in the pellet was counted in a gamma counter. Urinary creatinine, determined by autoanalyser, was used to normalize the urinary TGF-β1 level. The final concentration of TGF-β1 was expressed as μg g$^{-1}$ creatinine. The coefficients of variation of intra-assay and interassay were 7.5% and 10.0% respectively.

### Serological examination

HBsAg, anti-HCV and α-fetoprotein (AFP) were tested with Ausria-II, second-generation HCV enzyme immunoassay (EIA) and α-feto RIABEAD (Abbott Laboratories, Chicago, IL, USA) respectively. For anti-HCV, reactive specimens were retested. Repeatedly reactive samples were tested with another second-generation anti-HCV immunoassay (UBI HCV EIA; United Biomedical, Lake Success, NY, USA), which incorporates synthetic peptides from the capsid and non-structural protein region as the solid-phase antigen. Only specimens reactive in all three tests were considered as anti-HCV positive. Conventional liver function tests and creatinine level were determined with an autoanalyser.

### Transcatheter arterial chemoembolization (TACE)

TACE was performed according to the procedure described previously (Hsieh et al, 1996). A complete TACE was defined as: (1) all feeding arteries including collateral vessels were completely embolized angiographically; (2) serum AFP level decreased after TACE by more than 75% compared with the pre-TACE level; and (3) > 50% reduction in tumour size as evaluated by image study (sonography or computerized tomography) 1 month after TACE (Hsieh et al, 1996).

### Follow-up

The starting time of survival analysis was the day of diagnosis of HCC. All prognostic variables were measured on that day. The survival and the date of death were determined in December 1994 by review of clinical records, telephone or by consultation of population registries.

### Statistical analysis

Survival was expressed as median ± standard error, whereas other continuous data were expressed as median with ranges in parentheses. The difference between the unpaired continuous variables was compared with the Mann–Whitney U-test or the Kruskal–Wallis

© Cancer Research Campaign 1997

**Figure 1** Boxplot of urinary TGF-β1 level in patients with HCC and cirrhosis and in healthy controls. The horizontal line inside the box represents the median level. The lower and upper boundary represent the 25th and the 75th percentile of data respectively. TGF-β1 level in HCC was higher than that in cirrhosis or controls (each $P = 0.0001$; Mann–Whitney *U*-test). TGF-β1 level in cirrhosis was higher than that in controls ($P = 0.0001$; Mann–Whitney *U*-test)

**Table 2** Risk for HCC evaluated by stepwise logistic regression analysis of the comparison between HCC patients and patients with cirrhosis alone[a]

| Variables | Regression coefficient | Standard error | *P*-value | Odds ratio (95% CI) |
|---|---|---|---|---|
| TGF-β1 | 0.082 | 0.020 | 0.0001 | 1.08 (1.04–1.12) |
| AFP | 0.064 | 0.020 | 0.001 | 1.06 (1.02–1.10) |
| ALT | –0.014 | 0.006 | 0.030 | 0.98 (0.97–0.99) |
| ALP | 0.038 | 0.013 | 0.005 | 1.03 (1.01–1.06) |

[a]Dependent variable: existence of HCC. Independent variables: urinary TGF-β1, serum AFP, sex, age, serum albumin, globulin, aspartic aminotransferase, ALT, bilirubin (direct and indirect), ALP and γ-glutamyl transpeptidase. HCC, hepatocellular carcinoma; AFP, α-fetoprotein; TGF-β1, transforming growth factor-β1. ALT, alanine aminotransferase; ALP, alkaline phosphatase; CI, confidence interval.

one-way analysis of variance when appropriate. The Spearman rank correlation was used to calculate the relationship between continuous variables. The difference between paired continuous data was compared with the Wilcoxon signed-rank test. Chi-square test with Yates' correction was used to compare differences between proportions. Stepwise logistic regression was used for multivariate analysis. Odds ratio (OR) with 95% confidence interval (95% CI) was used to estimate causal relations between risk factors and exposure. The cumulative survival rate was determined using the product-limited (Kaplan–Meier) estimate (Kaplan and Meier, 1958), and a comparison of survival curves was made using the log-rank test. For evaluating the diagnostic performance of AFP and TGF-β1, we calculated the sensitivity, specificity, positive and negative predictive value, positive and negative likelihood ratio and diagnostic accuracy according to previous methods (Sox et al, 1989). The recommended diagnostic cut-off value of AFP was 400 ng $ml^{-1}$ (Sherlock and Dooley, 1993; Colombo, 1995). For TGF-β1, we used the receiver operating characteristic (ROC) curve to select the optimal cut-off value (Swets, 1988). The ROC curve was constructed by calculating the sensitivity and specificity of the TGF-β1 assay at several cut-off points. The cut-off value with the highest accuracy was selected as the diagnostic cut-off point. If more than one cut-off value showed the same accuracy, the cut-off value with nearly equal sensitivity and specificity was chosen. The differences in diagnostic accuracy between the marker tests were measured using McNemar's $\chi^2$-test. Two-tailed *P*-values and 95% CI were given when appropriate. An alpha of 0.05 was used as the indicator of statistical significance.

### RESULTS

### Urinary TGF-β1 and serum AFP levels in patients and healthy controls

The recovery of the acid precipitation method for urinary TGF-β1 was greater than 85% (data not shown). As shown in Figure 1, the urinary TGF-β1 level in patients with HCC (median 61.1, range 3.5–184.0 μg $g^{-1}$ creatinine) was significantly higher than in cirrhotic patients alone (median 30.3, range 4.3–52.5 μg $g^{-1}$ creatinine; $P = 0.0001$) or in healthy controls (median 12.2, range 1.5–33.6 μg $g^{-1}$ creatinine; $P = 0.0001$). The median level of urinary TGF-β1 in patients with cirrhosis alone was also statistically higher than that of healthy controls ($P = 0.0001$).

The upper limit of normal urinary TGF-β1 level was defined as values greater than the 95th percentile of healthy controls (32.3 μg $g^{-1}$ creatinine). Raised urinary TGF-β1 levels were noted in 58 (61.7%) of HCC patients and 46 (48.9%) of patients with cirrhosis alone. There is an inverse correlation between TGF-β1 and logAFP ($r = -0.292$, $P = 0.004$).

The upper limit of normal AFP level was defined as 20 ng $ml^{-1}$ (Sherlock and Dooley, 1993), whereas the recommended diagnostic cut-off value for HCC was 400 ng $ml^{-1}$ (Colombo, 1995). Serum AFP level less than 20 ng $ml^{-1}$ was noted in all healthy controls, 81 (86.1%) patients with cirrhosis alone and 33 (35.1%) patients with HCC. There were 45 (47.8%) HCC patients with AFP levels greater than 400 ng $ml^{-1}$ (Table 1). As shown in Table 1, the median level of serum AFP in patients with HCC was higher than that in cirrhotic patients alone ($P = 0.0001$) or in healthy controls (median 4, range 3–10 ng $ml^{-1}$; $P = 0.0001$).

### Association between levels of TGF-β1 and AFP and presence of HCC

Multivariate analysis was used to adjust for the possible confounding effects of sex, age and impaired liver function tests on the levels of urinary TGF-β1 and serum AFP in patients with HCC. Both TGF-β1 (OR 1.08, 95% CI 1.04–1.12, $P = 0.001$) and AFP (OR 1.06, 95% CI 1.02–1.10, $P = 0.001$) were found to be associated, in a dose-related fashion, with an increased risk for the presence of HCC (Table 2).

### TGF-β1 and AFP as tumour markers for HCC

The selected optimal cut-off value using the ROC curve was 50 μg $g^{-1}$ creatinine for TGF-β1. TGF-β1 levels greater than selected cut-off points were found in 53.1% (50 of 94) of patients with HCC, 1.1% (1 of 94) of patients with cirrhosis alone and none of the healthy controls. This cut-off value gave a specificity of 98.9% at sensitivity of 53.1%. The calculated diagnostic accuracy, positive and negative predictive values and positive and negative

© Cancer Research Campaign 1997

**Figure 2** Urinary TGF-β1 level before and after complete treatment with transcatheter arterial chemoembolization in HCC patients ($P = 0.0001$; Wilcoxon signed-rank test). The vertical bar indicates the median level of TGF-β1. TACE: transcatheter arterial chemoembolization

**Figure 3** Cumulative survival curves using the status of urinary TGF-β1 levels in patients with HCC. Increased TGF-β1 levels were defined as those values greater than the 95th percentile of healthy controls. The median survival in 58 patients with raised TGF-β1 levels (–●–) was shorter than in 36 patients with normal TGF-β1 levels (–■–) ($P = 0.018$; Kaplan–Meier method with log-rank test)

**Table 3** Urinary TGF-β1 level in relation to clinical features in HCC patients

| Parameters | Group | *n* | TGF-β1 (µg g$^{-1}$ creatinine) | *P*-value |
|---|---|---|---|---|
| Sex | | | | |
| | Male | 76 | 58.6 (3.5–164.3)[a] | NS |
| | Female | 18 | 66.4 (5.0–184.0) | |
| Age (years) | | | | |
| | < 40 | 14 | 69.7 (12.0–164.3) | NS |
| | ≥ 40 | 80 | 58.6 (3.5–184.0) | |
| Child–Pugh | | | | |
| | A | 36 | 23.7 (5.0–105.0)[b,c] | 0.0001 |
| | B | 38 | 55.1 (5.0–184.0)[b,d] | |
| | C | 20 | 123.7 (3.5–182.5)[c,d] | |
| HBsAG/anti-HCV | | | | |
| | Neg./neg. | 10 | 30.8 (5.0–98.4) | NS |
| | Pos./neg. | 58 | 63.2 (5.0–182.5) | |
| | Neg./pos. | 17 | 45.8 (3.5–184.0) | |
| | Pos./pos. | 9 | 65.3 (11.0–117.9) | |
| Metastasis | | | | |
| | Yes | 11 | 30.2 (8.0–142.3) | NS |
| | No | 83 | 63.4 (3.5–184.0) | |
| PVT | | | | |
| | Yes | 30 | 59.1 (5.0–182.5) | NS |
| | No | 64 | 61.1 (3.5–184.0) | |
| Location | | | | |
| | Single lobe | 52 | 33.2 (5.0–164.3) | 0.004 |
| | Multiple lobes | 42 | 67.9 (3.5–184.0) | |
| AFP | | | | |
| | ≤ 20 ng ml$^{-1}$ | 33 | 66.4 (6.0–184.0) | 0.024 |
| | > 20 ng ml$^{-1}$ | 61 | 36.4 (3.5–153.0) | |
| Clinical stage | | | | |
| | I | 10 | 17.5 (6.0–107.0)[e] | |
| | II | 43 | 61.8 (5.0–124.0)[f] | |
| | III | 10 | 65.3 (5.0–164.3) | |
| | IVA | 17 | 70.2 (3.5–187.4)[e,f] | |
| | IVB | 14 | 41.8 (6.0–142.3) | |

[a]Data are expressed as median with ranges in parentheses. [b]$P = 0.027$ (Mann–Whitney *U*-test). [c,d]$P = 0.0001$ (Mann–Whitney *U*-test). [e]$P = 0.01$ (Mann–Whitney *U*-test). [f]$P = 0.02$ (Mann–Whitney *U*-test). HCC, hepatocellular carcinoma; HBsAg, hepatitis B surface antigen; PVT, portal vein thrombosis; anti-HCV, antibodies to hepatitis C virus; pos., positive; neg., negative.

© Cancer Research Campaign 1997

**Table 4** Urinary TGF-β1 levels in relation to echographic type and tumour size

| Type and size of HCC | | TGF-β1 (μg g$^{-1}$ creatinine) |
|---|---|---|
| Diffuse | ($n$ = 30) | 96.5 (3.5–184.0)[a,b] |
| Non-diffuse | ($n$ = 64) | 33.3 (5.0–164.3)[b] |
| < 3 cm | ($n$ = 19) | 17.0 (5.0–112.3)[c,d] |
| 3–5 cm | ($n$ = 24) | 36.4 (5.0–143.3)[c,e] |
| > 5 cm | ($n$ = 21) | 56.9 (5.0–164.3)[d,e] |

[a]Data were expressed as median with ranges in parentheses. [b]$P$ = 0.001 (Mann–Whitney $U$-test). [c,d,e]$P$ < 0.02 (Kruskal–Wallis one-way analysis of variance). HCC, hepatocellular carcinoma; TGF-β1, transforming growth factor β1.

likelihood ratios were 76.0%, 98.0% and 67.8%, and 48.2 and 0.47 respectively. On the other hand, the recommended diagnostic level of AFP for HCC was 400 ng ml$^{-1}$ (Colombo, 1995). Using this cut-off value, the sensitivity was 47.8% with a specificity of 100%. The calculated diagnostic accuracy, positive and negative predictive values and positive and negative likelihood ratios were 73.9%, 100% and 65.7%, and 47.8 and 0.52 respectively. No matter which marker was used, there was no statistically significant difference between diagnostic accuracies.

When both AFP and TGF-β1 were determined in parallel, 30 (61.2%) of 49 HCC patients with AFP < 400 ng ml$^{-1}$ could be diagnosed. Both the sensitivity (79.7%) and diagnostic accuracy (81.3%) increased without decreasing the specificity (98.9%). The resulting positive and negative likelihood ratios were 74.5 and 0.2 respectively. It is of note that the diagnostic accuracy of using both AFP and TGF-β1 as markers was significantly higher than when using either marker alone ($P$ < 0.001; McNemar's $\chi^2$-test).

### Urinary TGF-β1 level before and after complete TACE

To assess the effect of therapy on the TGF-β1 level, we randomly chose 10 HCC patients with raised TGF-β1 levels for comparison. All these patients were defined as having complete TACE after therapy. As shown in Figure 2, the median urinary TGF-β1 level after TACE (45.5 μg g$^{-1}$ creatinine) was significantly lower than that (100.5 μg g$^{-1}$ creatinine) before TACE ($P$ = 0.0001; Wilcoxon signed-rank test). In another 10 patients without complete TACE, there was no significant difference in the TGF-β1 level before and after TACE (data not shown). Regardless of whether the TACE treatment was effective, TGF-β1 level increased as tumours recurred or increased in size during follow-up (data not shown).

### Urinary TGF-β1 level in relation to HCC survival

As shown in Figure 3, the median survival (169 ± 21, 95% CI 128–210 days) in 36 patients with normal TGF-β1 levels was significantly longer than that (86 ± 23, 95% CI 40–132 days) in 58 patients with elevated TGF-β1 levels ($P$ = 0.018, Kaplan–Meier method with log-rank test).

### Clinical relevance of urinary TGF-β1 level in patients with HCC

As shown in Table 3, the TGF-β1 levels in patients with Child–Pugh C were higher than those in Child–Pugh B or Child–Pugh A (each $P$ = 0.0001). Significantly elevated TGF-β1 levels were found in patients with an area of tumour involvement of more than one lobe ($P$ = 0.004). TGF-β1 levels in patients with normal AFP levels (≤ 20 ng ml$^{-1}$) were higher than those in patients with higher AFP levels ($P$ = 0.024; Table 3). Compared with patients with non-diffuse HCC, patients with diffuse HCC had higher urinary TGF-β1 levels ($P$ = 0.001; Table 4). Among non-diffuse HCC, TGF-β1 levels in patients with tumour size < 3 cm were lower than those with larger tumour size ($P$ < 0.02; Table 4). There was a positive correlation between TGF-β1 level and clinical stage of patients with HCC ($r$ = 0.178, $P$ < 0.05). As shown in Table 3, TGF-β1 levels in HCC patients with stage IVA were higher than in patients with stage I ($P$ = 0.01) or in patients with stage II ($P$ = 0.02). There was no statistical difference in TGF-β1 level with regard to sex, age, status of HBsAg and anti-HCV, extrahepatic metastasis or portal vein thrombosis (Table 3).

### DISCUSSION

Urinary TGF-β1 in man may reflect normal excretion of the polypeptide by the urinary tract. It could also reflect the production of TGF-β1 elsewhere in the body and being filtered by the glomeruli or being produced lower in the urinary tract. Although we did not determine plasma TGF-β1 level, there was no relationship between plasma TGF-β level and renal function (Coupes et al, 1994). In this study, we still use urinary creatinine to normalize the TGF-β1 concentration. It is worth noting that the larger the tumour size, the higher the urinary TGF-β1 level (Table 4). Moreover, the significantly lower urinary TGF-β1 level after complete TACE therapy (Figure 2) suggested that TGF-β1 level might be related to tumour mass. Decreased plasma TGF-β1 level after successful anti-cancer treatment has also been reported previously (Shirai et al, 1992, 1994). These observations indicated that both urinary and plasma TGF-β1 levels might reflect liver tissue TGF-β1.

Liver is the major site of clearance and metabolism of biologically active TGF-β1 (Roberts and Sporn, 1990; Fausto et al, 1991). Raised TGF-β1 level may be caused by increased production and/or decreased clearance. An increased TGF-β1 production has been reported after hepatectomy and in some liver disease (Ito et al, 1990, 1991; Roberts and Sporn, 1990; Shirai et al, 1992, 1994; Bedossa et al, 1995). In this study, the raised TGF-β1 levels in patients with cirrhosis alone might be caused by impaired liver function (Figure 1). Elevated urinary TGF-β1 levels in patients with cirrhotic HCC might be due to decreased clearance and/or increased production. The association between raised TGF-β1 level and worse Child–Pugh grades in patients with cirrhotic HCC (Table 3) and patients with cirrhosis alone (data not shown) suggested the contribution of impaired liver function. After adjusting for the possible confounding effects caused by impaired liver function, our results indicate that urinary TGF-β1 level is significantly associated, in a dose-related fashion, with the presence of HCC (Table 2). In addition, larger tumours were frequently associated with higher TGF-β1 levels (Table 4). The significantly decreased TGF-β1 levels after complete TACE treatment in patients with HCC (Figure 2) also implies that TGF-β1 might be related to tumour mass and that raised TGF-β1 levels in HCC were caused by increased production. On the other hand, the inverse relationship between raised urinary TGF-β1 level and survival (Figure 3) suggests that TGF-β1 might be a predictor of poor prognosis of HCC.

© Cancer Research Campaign 1997

How TGF-β is involved in the growth control of HCC remains unclear. Given the inhibitory effects of TGF-β in the normal and neoplastic hepatocytes, it is logical to expect that absence of the factor or a loss of sensitivity to TGF-β could contribute to cell transformation (Fausto et al, 1991). However, the discrepancy between the increase in TGF-β level in HCC and the highly proliferating cell rate in HCC suggests that HCC cells have lost autocrine growth inhibition of TGF-β during malignant transformation (Roberts et al, 1988; Roberts and Sporn, 1990; Bedossa et al, 1995). The escape of tumoral hepatocytes from the control of cell mito-inhibition by TGF-β1, despite its overexpression by these cells, might be related to secretion of biologically inactive or latent TGF-β and to the absence of or lower numbers of TGF-β receptors on the plasma membrane of malignant hepatocytes (Roberts et al, 1988; Fausto et al, 1991; Bedossa et al, 1995). Increased secretion of TGF-β by cancer cells that have lost responsiveness to its growth inhibitory activities is thought to facilitate tumour progression by indirect means, such as suppression of immune surveillance and stimulation of tumour stroma (Roberts et al, 1988; Roberts and Sporn, 1990; Fausto, 1991; Fausto et al, 1991). Tumour cells might indirectly support their growth by paracrine action of the TGF-β on the supporting stromal elements. By extracellular matrix formation and angiogenesis, the consequent neovascularization removes the limitations of diffusion through solid tissues (Roberts et al, 1988; Fausto, 1991). This in turn results both in a rapid increase in tumour size and provision of a route for metastasis throughout the body. Moreover, TGF-β not only stimulates the synthesis of tumour stromal elements but also creates a selective environment in which 'partly transformed' cells with defective TGF-β response can proliferate and form tumours (Roberts et al, 1988; Fausto, 1991). In terms of a model for TGF-β action in carcinogenesis, it is known that most tumour cells express TGF-β mRNA and that many secrete TGF-β (Ito et al, 1990, 1991; Fausto, 1991; Fausto et al, 1991; Shirai et al, 1992, 1994; Bedossa et al, 1995). Such growth factor secretion might result in stimulation of the growth of the tumour with accompanying stimulation of the development of supporting tumour stromal elements. On the other hand, although we give no indication of cellular source of TGF-β in our patients, the cellular source of TGF-β in patients with cirrhotic HCC has been well studied by Bedossa et al (1995). Besides in HCC cells, TGF-β mRNA and TGF-β did exist in extracellular matrix along the fibrous septa. Hence, TGF-β may actually derive from tumour stroma and thus may be an indicator of tumour desmoplasia or may correlate with tumour necrosis.

HCC appears to be associated with hepatitis B and C viral infection and is common in patients with cirrhosis caused by chronic viral hepatitis (Jeng and Tsai, 1991; Tsai et al, 1994a–c, 1996). Cirrhosis is considered to be a premalignant lesion of HCC (Sherlock and Dooley, 1993; Tsai et al, 1994a–c, 1996); thus, it is important to diagnose HCC in cirrhotic patients. Among various serological markers developed for diagnosis of HCC, AFP is one of the most intensively studied tumour markers (Sherlock and Dooley, 1993; Chuang et al, 1994; Colombo, 1995; Tsai et al, 1995). As shown in this study, an AFP level less than the recommended diagnostic level (400 ng $ml^{-1}$) was noted in 52.1.% (49 of 94) of HCC patients at the time of tumour detection. Furthermore, at least one-third of small HCC and up to 30% of advanced HCC will be missed unless other diagnostic tools are used (Sherlock and Dooley, 1993; Tsai et al, 1994*b*, 1995; Colombo, 1995). In addition, AFP may be elevated in non-malignant liver disease (Sherlock and Dooley, 1993; Colombo, 1995; Tsai et al, 1995). It is obvious that AFP alone is not a reliable indicator for the detection of HCC in patients with a low AFP value. On the other hand, the main problem regarding the diagnosis of HCC is that of detection of tumour presence in those cirrhotic patients in whom the AFP is raised but diagnostic. Therefore, additional and more sensitive diagnostic tools must be sought.

In this study, we have demonstrated that urinary TGF-β1 might be used as a tumour marker for HCC. After comparing the diagnostic performance of AFP and TGF-β1, either marker showed a good specificity, moderate sensitivity and high positive likelihood ratio. There was no significant difference between their diagnostic accuracies. Moreover, determination of AFP and TGF-β1 in parallel significantly improved the diagnostic accuracy and sensitivity without decreasing the specificity. Although each test may not have sufficient sensitivity, the simultaneous use of both tests may be highly discriminatory in the detection of HCC. However, parallel detection of both markers increases the number of tests performed, which is likely to have cost implications. Hence, we suggest that assay for urinary TGF-β1 should be performed to improve the detection of HCC with low AFP production. It is noteworthy that AFP is an oncofetal protein produced by HCC. Although the AFP gene was re-expressed in hepatoma cells, TGF-β1 may repress the AFP gene expression in hepatoma cells (Nakao et al, 1991). Our results also show a reverse relationship between levels of serum AFP and urinary TGF-β1. The urinary TGF-β1 level in HCC patients with normal AFP level was statistically higher than that in patients with raised AFP (Table 3). This significant inverse trend still existed even when the higher cut-off value of AFP (100 or 400 ng $ml^{-1}$) was used (data not shown). This observation also favours the use of urinary TGF-β1 as a complementary tumour marker for the detection of HCC in AFP-non-producing tumours.

In conclusion, this study shows that urinary TGF-β1 level increases in patients with cirrhotic HCC. Raised urinary TGF-β1 level can be used as a tumour marker for HCC. It is also a predictor of poor survival in HCC.

### ACKNOWLEDGEMENT

This study was supported by a grant from the National Science Council of the Republic of China (NSC 82-0412-B-037-003).

### REFERENCES

American Joint Committee on Cancer (1992) *Manual for Staging of Cancer*. JB Lippincott: Philadelphia

Bedossa P, Peltier E, Terris B, Franco D and Poynard T (1995) Transforming growth factor-beta 1 (TGF-β1) and TGF-β1 receptors in normal, cirrhotic, and neoplastic human livers. *Hepatology* **21**: 760–766

Bissell DM and Maher JJ (1996) Hepatic fibrosis and cirrhosis. In *Hepatology: a Textbook of Liver Disease*, Zakim D and Boyer TD. (eds), pp. 506–525, WB Saunders: Philadelphia

Chuang LY, Tsai JH, Yeh YH, Chang CC, Yeh HW, Guh JY and Tsai JF (1991) Epidermal growth factor-related transforming growth factors in the urine of patients with hepatocellular carcinoma. *Hepatology* **13**: 1112–1116

Chuang LY, Hon WC, Yang ML, Chang CC and Tsai JF (1994) Urinary epidermal growth factor receptor-binding growth factors in the tumors of the digestive tract. *Clin Biochem* **27**: 485–489

Colombo M (1995) Should patients with chronic viral hepatitis be screened for hepatocellular carcinoma? *Viral Hepatitis Rev* **1**: 67–75

Coupes BM, Newstead CG, Short CD and Brenchley PEC (1994) Transforming growth factor β1 in renal allograft recipients. *Transplantation* **57**: 1727–1731

© Cancer Research Campaign 1997

Fausto N (1991) Multifunctional roles of transforming growth factor beta 1. *Lab Invest* **65**: 497–499

Fausto N, Mead JE, Gruppuso PA and Braun L (1991) TGF-β in liver development, regeneration, and carcinogenesis. *Ann NY Acad Sci* **593**: 231–242

Hsieh MY, Chen SC, Lu SN, Wang LY, Tsai JF, Chuang WL, Lin ZY and Chang WY (1996) Treatment of hepatocellular carcinoma smaller than 5 cm by transcatheter arterial chemoembolization. *Kaohsiung J Med Sci* **12**: 274–278

Ito N, Kawata S, Tamura S, Takaishi K, Yabuuchi I, Matsuda Y, Nishioka M and Tarui S (1990) Expression of transforming growth factor-beta 1 mRNA in human hepatocellular carcinoma. *Jpn J Cancer Res* **81**: 1202–1205

Ito N, Kawata S, Tamura S, Takaishi K, Shirai Y, Kiso S, Yabuuchi I, Matsuda Y, Nishioka M and Tarui S (1991) Elevated levels of transforming growth factor-beta and its polypeptide in human hepatocellular carcinoma. *Cancer Res* **15**: 4080–4083

Jeng JE and Tsai JF (1991) Hepatitis C virus antibody in hepatocellular carcinoma in Taiwan. *J Med Virol* **34**: 74–77

Kaplan EL and Meier P (1958) Nonparametric estimation from incomplete observation. *Am Stat Assoc* **53**: 457–458

Lee GH, Merlino G and Fausto N (1992) Development of liver tumors in transforming growth factor α transgenic mice. *Cancer Res* **52**: 5162–5170

Nakao K, Nakata K, Mitsuoka S, Ohtsuru A, Ido A, Hatano M, Sato Y, Nakayama T, Shima M, Kusumoto Y, Koji T, Tamaoki T and Nagataki S (1991) Transforming growth factor β1 differentially regulates α-fetoprotein and albumin in HuH-7 human hepatoma cells. *Biochem Biophys Res Commun* **174**: 1294–1299

Nishimura R, Okumura H, Noda K, Yasumitsu H and Umeda M (1986) High level of β type transforming growth factor activity in human urine obtained from cancer patients. *Jpn J Cancer Res* **77**: 560–567

Pugh RN, Murray-Lyon IM, Dawson JL, Peitroni MC and Williams R (1973) Transection of the esophagus for bleeding esophageal varices. *Br J Surg* **60**: 646–649

Ranganathan G, Lyons R, Jiang NS and Moses H (1987) Transforming growth factor β in normal human urine. *Biochem Biophys Res Comm* **148**: 1503–1512

Roberts AB and Sporn MB (1990) The transforming growth factor betas. In *Handbook of Experimental Pharmacology*, Sporn MB and Roberts AB. (eds), Vol. 95, pp. 419–472. Springer: Heidelberg, Germany

Roberts AB, Thompson NL, Heine U, Flanders C and Sporn MB (1988) Transforming growth factor-beta: possible roles in carcinogenesis. *Br J Cancer* **57**: 594–600

Sherlock S and Dooley J (1993) *Disease of the Liver and Biliary System.* pp. 503–531. Blackwell Scientific: Oxford

Sherwin SA, Twardzik DR, Bohn WH, Cockley KD and Todaro GJ (1983) High-molecular-weight transforming growth factor activity in the urine of patients with disseminated cancer. *Cancer Res* **43**: 403–407

Shirai Y, Kawata S, Ito N, Tamura S, Takaishi K, Kiso S, Tsushima H and Matsuzawa Y (1992) Elevated levels of plasma transforming growth factor-β in patients with hepatocellular carcinoma. *Jpn J Cancer Res* **83**: 676–679

Shirai Y, Kawata S, Tamura S, Ito N, Tsushima H, Takaishi K, Kiso S and Matsuzawa Y (1994) Plasma transforming growth factor-β1 in patients with hepatocellular carcinoma. *Cancer* **73**: 2275–2279

Sox HC, Blatt MA, Higgins MC and Marton K (1989) *Medical Decision Making.* pp. 67–146. Butterworth: London

Swets JA (1988) Measuring the accuracy of diagnostic systems. *Science* **240**: 1285–1293

Tsai JF, Chang WY, Jeng JE, Ho MS, Wang LY, Hsieh MY, Chen SC, Chuang WL Lin ZY and Tsai JH (1993) Hepatitis C virus infection as a risk factor for non-alcoholic liver cirrhosis in Taiwan. *J Med Virol* **41**: 296–300

Tsai JF, Chang WY, Jeng JE, Ho MS, Lin ZY and Tsai JH (1994*a*) Hepatitis B and C virus infection as risk factors for liver cirrhosis and cirrhotic hepatocellular carcinoma: a case–control study. *Liver* **14**: 98–102

Tsai JF, Chang WY, Jeng JE, Ho MS, Lin ZY and Tsai JH (1994*b*) Frequency of raised alpha-fetoprotein level among Chinese patients with hepatocellular carcinoma related to hepatitis B and C. *Br J Cancer* **69**: 1157–1159

Tsai JF, Chang WY, Jeng JE, Ho MS, Lin ZY and Tsai JH (1994*c*) Hepatitis B and C virus infection as risk factors for hepatocellular carcinoma in Chinese: a case–control study. *Int J Cancer* **56**: 619–621

Tsai JF, Jeng JE, Ho MS, Chang WY, Lin ZY and Tsai JH (1995) Clinical evaluation of serum α-fetoprotein and circulating immune complexes as tumor markers of hepatocellular carcinoma. *Br J Cancer* **72**: 442–446

Tsai JF, Jeng JE, Ho MS, Chang WY, Hsieh MY, Lin ZY and Tsai JH (1996) Additive effect modification of hepatitis B surface antigen and e antigen on the development of hepatocellular carcinoma. *Br J Cancer* **73**: 1498–1502

Yeh YC, Tsai JF, Chuang LY, Yeh HW, Tsai JH, Florine DL and Tam JP (1987) Elevation of transforming growth factor α and its relationship to the epidermal growth factor and α-fetoprotein levels in patients with hepatocellular carcinoma. *Cancer Res* **47**: 896–901

© Cancer Research Campaign 1997